# Hydrogen Release From Silicon─Hydrogen Systems: From Surface Chemistry to Higher‐Coordinate Molecular Mechanisms

**DOI:** 10.1002/chem.71612

**Published:** 2026-08-31

**Authors:** Robin Rothfelder, Jonathan O. Bauer

**Affiliations:** ^1^ Faculty of Chemistry and Pharmacy, Institute of Inorganic Chemistry University of Regensburg Regensburg Germany

**Keywords:** dehydrogenation, higher‐coordinate silicon species, hydrogen release mechanisms, Si─H bond activation, silicon

## Abstract

Hydrogen release from silicon–hydrogen systems spans a field ranging from extended silicon materials to discrete molecular compounds. Across these regimes, Si─H activation and H_2_ evolution arise from the interplay of structure, coordination environment, and access to reactive geometries. In extended silicon systems, hydrogen release is governed by structure‐dependent recombinative desorption processes. Zintl phases bridge solid‐state and molecular silicon chemistry by enabling transfer of polyanionic silicon units into solution, thereby providing access to discrete Si─H reactivity. In molecular systems, metal‐mediated and metal‐free pathways contribute to hydrogen release, including oxidative addition, σ‐bond metathesis, electrophilic activation, cooperative heterolysis, and donor‐assisted Si─H activation. These mechanistic manifolds form a continuum in which variations in coordination environment and electronic structure determine whether dehydrocoupling, hydrogen transfer, or bond‐forming processes dominate. A recurring mechanistic feature is the involvement of higher‐coordinate silicon species. However, productive H_2_ evolution depends on their formation, but also on the ability of the silicon framework to access reactive geometries through fluxional behavior and dynamic permutational isomerism. Overall, silicon‐based hydrogen‐release chemistry illustrates how structure, coordination, and molecular dynamics collectively govern bond activation and hydrogen evolution, providing a conceptual basis for the design of silicon‐based hydrogen storage and release systems.

## Graphical abstract

Hydrogen‐release processes in silicon chemistry occur across a broad spectrum of systems, including crystalline silicon surfaces, nanoporous materials, Zintl phases, and molecular hydrides. This Review highlights how Si─H activation and H_2_ evolution are governed by structure, coordination environment, and dynamic access to reactive geometries, with particular emphasis on higher‐coordinate silicon species and fluxional hydrogen‐release pathways.

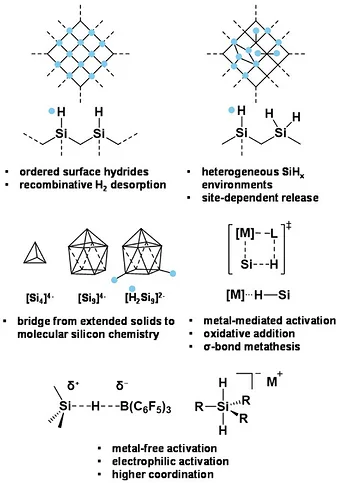

## 1 Introduction

The transition from fossil‐based to renewable energy systems has intensified the search for efficient and safe energy‐storage technologies. In this context, molecular hydrogen (H_2_) has emerged as both an important chemical energy carrier and a valuable industrial feedstock [1, 2, 3, 4]. However, its widespread implementation remains constrained by challenges associated with efficient storage, safe transport with minimal losses, and the controlled release of stored hydrogen [1, 2, 3, 4]. Although conventional physical storage methods such as cryogenic liquefaction and high‐pressure compression are technically feasible, they are accompanied by considerable safety concerns, substantial infrastructural demands, and significant energy penalties [1, 2, 3, 4]. As a result, growing attention has shifted toward chemical hydrogen‐storage strategies, particularly liquid organic hydrogen carriers (LOHCs) [5, 6, 7, 8, 9, 10].

Although LOHC systems offer significant logistical advantages, particularly because they are often compatible with existing liquid‐fuel infrastructure, their broader application remains limited by the high energy demand of the dehydrogenation step, the requirement for efficient catalyst systems, and the formation of degradation products during repeated cycling [5, 6, 7, 8, 9, 10]. In addition, the environmental and toxicological properties of potential carrier compounds must be assessed individually rather than generalized across an entire class of materials [11]. Against this background, increasing attention has been directed toward alternative hydrogen‐storage concepts that may circumvent at least some of these limitations [12, 13, 14, 15, 16, 17]. Among these, silicon‐based systems have attracted particular interest [12, 13, 14, 15, 16, 17, 18]. This broad class includes hydrogen‐terminated silicon surfaces and nanostructured silicon materials, as well as hydrogenated amorphous silicon and discrete molecular silicon hydrides (Figure 1) [18, 19, 20, 21, 22, 23, 24, 25, 26, 27, 28]. Their appeal arises not only from the abundance of silicon, but more importantly from the fundamentally distinct chemical behavior of Si─H bonds compared to the C─H bonds that dominate conventional LOHC systems [18, 19]. At the same time, silicon‐based hydrogen‐release systems must be evaluated with the same level of scrutiny as any other carrier platform, since their safety, stability, and sustainability strongly depend on molecular structure, reaction conditions, and the underlying structural motif [11, 18].

**FIGURE 1:**
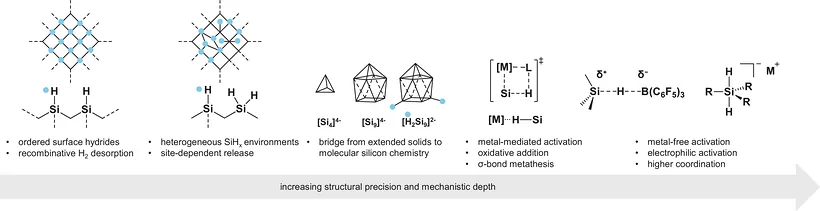
Structural and mechanistic landscape of silicon‐based hydrogen‐release chemistry. The field encompasses hydrogen‐terminated crystalline surfaces, nanoporous and amorphous silicon materials, Zintl‐derived cluster chemistry, and discrete molecular systems. Across this continuum, hydrogen release evolves from surface‐ and environment‐controlled desorption processes in extended materials to increasingly well‐defined bond‐activation pathways in molecular systems, including metal‐mediated and metal‐free electrophilic or higher‐coordinate silicon manifolds.

### 1.1 Electronic Structure and Reactivity of Si─H Bonds

The interest in silicon‐based compounds for hydrogen‐release chemistry arises from the distinct electronic and thermodynamic properties of the Si─H bond [18, 19]. In contrast to the largely nonpolar C─H bonds found in alkanes, Si─H bonds are intrinsically polarized because silicon is less electronegative than hydrogen. Consequently, the hydrogen atom in hydrosilanes exhibits pronounced hydridic character [18, 19]. This polarization is of fundamental importance because it enables reaction pathways and activation modes that are far less common in conventional carbon‐based hydrogen carriers [18, 19, 27, 28]. Silicon hydrides therefore differ not only in reactivity, but also in the mechanistic principles by which hydrogen release can be accomplished [18, 19, 27, 28].

Another important feature is the comparatively low bond strength of many Si─H bonds relative to typical alkane C─H bonds [18, 19]. This difference is commonly attributed to the larger size of silicon, the longer Si─H bond distance, and less efficient orbital overlap compared to carbon‐based analogues [18, 19]. As a result, Si─H bond activation often proceeds under substantially milder conditions than the activation of conventional C─H bonds [18, 19]. Together, these characteristics make silicon hydrides highly attractive motifs for hydrogen‐storage and hydrogen‐release chemistry [12, 13, 14, 15, 16, 17, 18, 19].

In addition to the extensive transition‐metal‐based literature on Si─H activation, more recent studies have highlighted metal‐free and main‐group approaches that exploit bond polarization, higher coordination, and ligand‐controlled reactivity [19, 27, 28]. It is precisely this interplay between intrinsic bond properties and tunable coordination chemistry that renders silicon‐based systems particularly promising for the development of alternative hydrogen‐release concepts [19, 27, 28].

### 1.2 Structural Landscape of Silicon‐Based Hydrogen Carriers

The structural landscape of silicon‐based systems relevant to hydrogen storage and release is remarkably diverse [12, 13, 14, 15, 16, 17, 18, 19, 20, 21, 22, 23, 24, 25, 26, 27, 28]. On the materials side, it encompasses hydrogen‐terminated silicon surfaces, porous and nanostructured silicon materials, as well as hydrogenated amorphous silicon networks. On the molecular level, it includes discrete silicon hydrides and related compounds (Figure 1) [18, 19, 20, 21, 22, 23, 24, 25, 26, 27, 28]. These classes differ fundamentally in the manner by which hydrogen is bound, activated, and ultimately released. Accordingly, the following chapters discuss extended silicon materials and molecular silicon systems separately [18, 19, 20, 21, 22, 23, 24, 25, 26, 27, 28].

## 2 Solid‐State and Surface Chemistry of Silicon‐Based Hydrides

The interaction of atomic and molecular hydrogen with elemental silicon has provided much of the fundamental basis for understanding Si─H bond formation, passivation, dissociation, and hydrogen release in extended silicon‐based systems [29, 30, 31, 32, 33]. At the same time, silicon–hydrogen chemistry varies substantially across different structural forms of elemental silicon and is strongly shaped by the underlying structural regime [29, 30, 33, 34, 35, 36]. From crystalline silicon surfaces to nanoporous and amorphous silicon materials and ultimately to Zintl phases that bridge extended and molecular silicon chemistry, both the local Si─H environments and the mechanistic principles of hydrogen binding, activation, and release change profoundly [29, 36, 37, 38]. Accordingly, solid‐state silicon hydride chemistry is best viewed not as a single, uniform field of reactivity, but rather as a structural continuum in which increasing disorder, surface accessibility, and electronic differentiation progressively redefine the behavior of the Si─H bond.

### 2.1 Hydrogenated Silicon Surfaces: Structure and Reactivity

The fundamental understanding of Si─H bonding in silicon–hydrogen chemistry is deeply rooted in solid‐state and surface chemistry [29, 30, 31, 32, 33, 39]. Crystalline silicon surfaces provide the most well‐defined reference systems for investigating these processes. In bulk crystalline silicon, each silicon atom is tetrahedrally coordinated by four Si─Si bonds, whereas surface formation inevitably generates undercoordinated atoms and dangling‐bond‐derived reactivity [29, 30, 31]. Adsorption of hydrogen passivates these reactive sites through Si─H bond formation and thereby fundamentally alters both the electronic structure and the chemical reactivity of the surface [29, 30, 32, 39].

Depending on crystal orientation, distinct surface geometries and bonding situations emerge [30, 39, 40]. Among the many possible surface terminations, Si(100) and Si(111) have been studied most extensively and therefore serve as the principal model systems for hydrogen adsorption, passivation, surface reconstruction, and hydrogen desorption [30, 39, 40, 41]. On Si(100), the surface reconstructs through formation of Si─Si dimers, thereby reducing the number of dangling bonds. As a consequence, the hydrogen chemistry of this surface is governed primarily by these dimer units rather than by isolated unsaturated silicon atoms (Figure 2) [40, 42].

**FIGURE 2:**
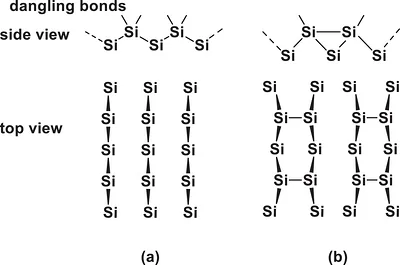
Structure of Si(100) showing the reconstruction from (a) 1 × 1 to (b) 2 × 1 Si─Si dimer formation.

By contrast, the reconstructed Si(111)‐(7 × 7) surface contains chemically distinct adatom and rest‐atom sites, such that hydrogen reactivity becomes highly site dependent [40, 42].

The conventional description of hydrogen‐terminated silicon surfaces derives mainly from studies involving atomic hydrogen [39, 40]. Under typical conditions, molecular hydrogen exhibits only a very low sticking probability on ideal silicon surfaces, and adsorption is therefore treated as an activated dissociative process closely related to the reverse process of recombinative H_2_ desorption [41, 43]. Mechanistically, this distinction is highly important because hydrogen release from silicon surfaces is not governed simply by cleavage of isolated Si─H bonds, but by structure‐dependent recombination pathways involving neighboring hydride sites, local geometry, and surface rearrangement during desorption [32, 33, 41, 43].

Hydrogen adsorption on crystalline silicon surfaces is furthermore not restricted to simple monohydride termination. At elevated hydrogen coverages and under suitable thermal conditions, more highly hydrogenated local surface motifs may form, particularly on Si(100), where dimer‐derived surface structures allow the formation of dihydride‐like configurations (Figure 3) [39, 40]. These species are best regarded as localized structural motifs rather than as a uniform description of the entire hydrogenated surface. This local character is one reason why hydrogen desorption from silicon surfaces is highly structure dependent. The feasibility and kinetics of H_2_ evolution depend not only on Si─H bond cleavage itself, but also on the local surface environment, the proximity of neighboring hydrides, and the extent to which the surface can reorganize during recombinative desorption [32, 33, 41, 43]. Although such processes may induce local roughening or restructuring, ordered crystalline silicon surfaces remain the most well‐defined systems for understanding hydrogen adsorption, passivation, and desorption in silicon‐based materials [29, 30, 32, 39].

**FIGURE 3:**
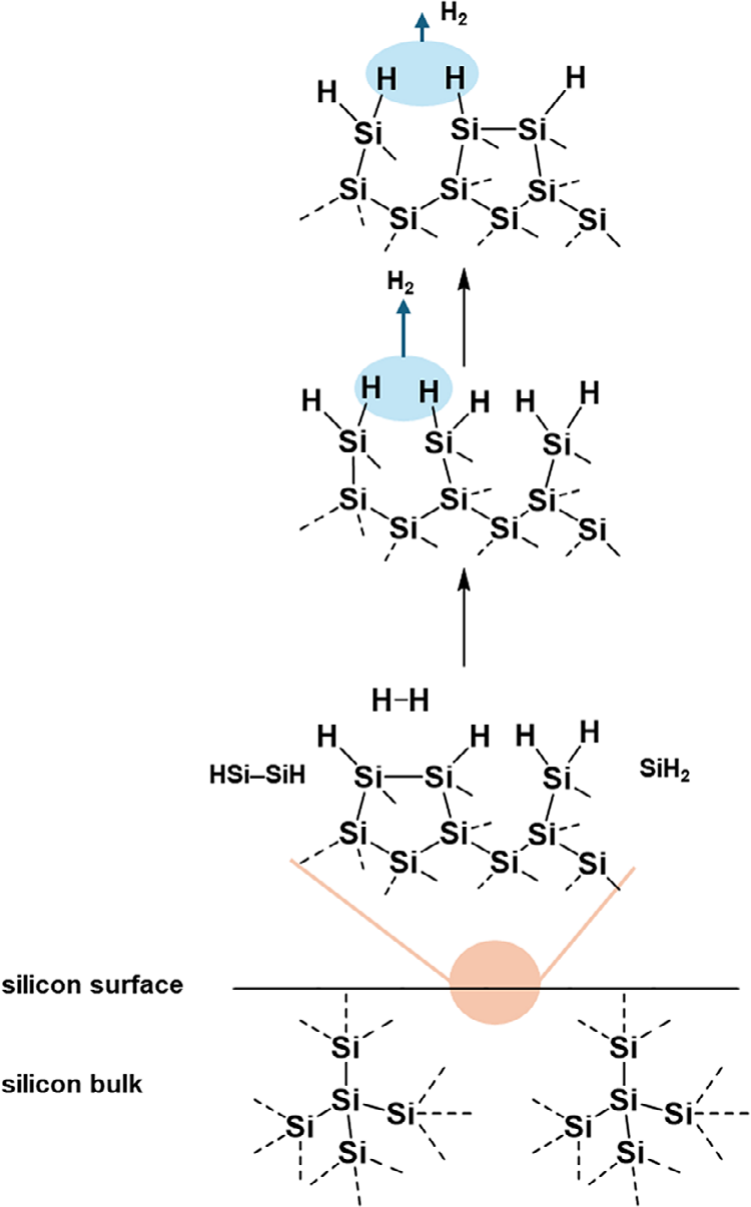
Ordered surface structure determines the local Si─H environments that can form upon hydrogen adsorption. At higher hydrogen coverage, especially on Si(100), more highly hydrogenated surface motifs such as SiH_2_ dihydride‐like species and HSi─SiH hydrogenated dimer arrangements can occur. Hydrogen desorption is not shown as simple cleavage of an isolated Si─H bond, but as a structure‐dependent recombinative H_2_ elimination process involving neighboring surface hydrides. Depending on the local geometry and hydride configuration, this process can be accompanied by surface rearrangement and local roughening of the crystalline surface.

The chemistry of the Si─H bond changes substantially along the transition from ordered crystalline model surfaces toward high‐surface‐area, structurally heterogeneous silicon materials. This heterogeneity becomes particularly pronounced in nanoporous silicon [20, 44, 45].

### 2.2 Nanoporous and Amorphous Silicon: Heterogeneous Hydrogen Environments

Compared with well‐defined crystalline model surfaces such as Si(100) and Si(111), the hydrogen chemistry of nanoporous silicon is governed by a far larger internal surface area and by a much broader distribution of local Si─H environments [20, 44, 45]. Nanoporous silicon is commonly generated by electrochemical anodization of bulk silicon in HF‐containing media (Figure 4). These etching processes produce a roughened silicon framework with an interconnected network of pores, channels, and tunnels, thereby exposing a wide variety of local surface environments. As a consequence, different Si─H motifs and bonding situations can coexist within the same material. In contrast to ideal crystalline model surfaces, hydrogen adsorption and desorption in nanoporous silicon are therefore controlled by structural heterogeneity, surface accessibility, and local geometry (Figure 5) [41, 44, 45, 46, 47]. Mixed populations of SiH, SiH_2_, and related surface species are thus not merely coverage‐dependent variants of one idealized surface model, but an intrinsic feature of the material itself [20, 44, 45, 48].

**FIGURE 4:**
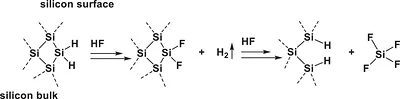
HF treatment of a silicon surface leading to hydrogen‐terminated pore walls.

**FIGURE 5:**
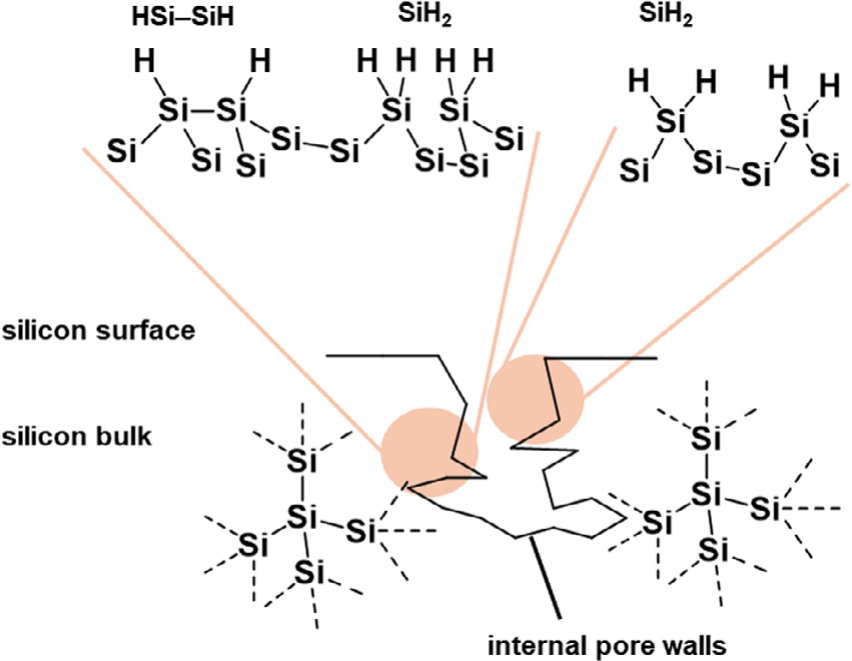
Schematic representation of nanoporous silicon with roughened internal surfaces and an interconnected network of pores and tunnels. The internal pore walls expose different local surface environments and Si─H motifs, giving rise to structurally heterogeneous hydrogen adsorption and desorption behavior.

Hydrogen desorption in nanoporous silicon is correspondingly heterogeneous [20, 45, 47, 48]. It depends on the local pore environment, surface accessibility, site type, thermal history, and the extent to which local reconstruction and recombinative H_2_ formation are possible. Hydrogen release is therefore not governed solely by cleavage of individual Si─H bonds, but rather by the combined influence of local SiH_x_ stability, neighboring‐site arrangement, diffusion and recombination pathways, and structural reorganization within the porous network [20, 23, 45, 47, 48, 49].

In this respect, nanoporous silicon is not simply a crystalline silicon surface with increased area, but a fundamentally surface‐dominated material in which architecture, confinement, and local heterogeneity become mechanistically decisive [45, 47, 49]. At the same time, hydrogen‐terminated porous silicon surfaces remain chemically versatile and can undergo subsequent surface transformations such as hydrosilylation, further emphasizing that nanoporous silicon functions not only as a hydrogen‐bearing material but also as a reactive silicon–hydride interface [46]. In contact with water or under oxidative conditions, this combination of high surface area, accessible surface hydrides, and structural flexibility can directly translate into hydrogen‐generation behavior in nanostructured silicon systems [23, 49].

A related but conceptually distinct limiting case is reached when long‐range crystalline order is lost entirely and amorphous silicon networks are formed [29, 30, 33, 34, 35, 36]. In amorphous silicon, hydrogen still serves to passivate dangling bonds, but the local environment is no longer defined by ideal crystalline surface geometries [29, 33, 36]. Instead, Si─H bond formation, dissociation, hydrogen transport, and hydrogen mobility occur across a distribution of local coordination environments and defect structures coupled to continuous network relaxation [33, 34, 35, 36]. Amorphous silicon therefore represents the limiting case of structural heterogeneity among extended silicon hydride environments. Although the chemistry remains fundamentally solid‐state in nature, the local Si─H motif can no longer be uniquely assigned to a single lattice‐derived geometry [29, 34, 35, 36].

Whereas nanoporous and amorphous silicon broaden the environmental diversity of silicon–hydrogen chemistry relative to the more ordered reference case of crystalline silicon surfaces, Zintl phases introduce a fundamentally different structural and electronic situation. They retain a solid‐state foundation while already approaching key characteristics of molecular silicon chemistry [37, 38].

### 2.3 Zintl Phases as Structural Bridge to Molecular Silicon Hydride Chemistry

Zintl phases introduce electronically distinct silicon frameworks that formally belong to solid‐state chemistry, yet simultaneously function as a structural and conceptual bridge toward molecular silicon chemistry [37, 38, 50]. In general, Zintl phases are intermetallic compounds composed of highly electropositive metals, typically alkali or alkaline‐earth metals, and more electronegative p‐block elements [37, 38, 51]. In silicon‐containing systems, electron transfer from the electropositive component to silicon gives rise to polyanionic silicon substructures. Depending on composition, electron count, and counterion size, these may occur as isolated clusters, layered arrangements, or extended anionic frameworks [37, 38, 50, 51].

Importantly, these polyanionic silicon units are already preorganized in the solid state and can, under suitable conditions, be transferred into solution and employed as reactive molecular precursors [37, 38, 51]. Representative examples include the tetrahedral [Si_4_]^4–^ cluster present in phases such as K_4_Si_4_ and K_12_Si_17_, as well as the [Si_9_]^4–^ cluster accessible from K_12_Si_17_ through extraction chemistry (Figure 6) [37, 38, 51, 52, 53]. Once transferred into solution, these silicon cluster systems no longer remain static. Studies on K_12_Si_17_‐derived Si_9_ chemistry revealed additional speciation processes, including reduced cluster derivatives such as [Si_9_]^3–^ and [Si_9_]^2–^ as well as protonated species including [HSi_9_]^3–^ and [H_2_Si_9_]^2–^ [52, 53]. The significance of these findings lies less in their preparative outcome than in the demonstration that Zintl‐derived silicon clusters can undergo genuine molecular‐type follow‐up chemistry, including protonation and controlled functionalization [51, 52, 53, 54]. This aspect is particularly relevant in the context of hydrogen‐release chemistry because it shows that solid‐state‐derived silicon frameworks can be transformed into discrete molecular species in which protonation and Si─H bond formation become chemically accessible. In this sense, Zintl phases are not hydrogen‐release systems in the same direct manner as the molecular hydrosilane‐based systems discussed later, but they establish the conceptual transition toward them. They demonstrate that silicon units originating from the solid state can evolve into solution‐accessible species in which Si─H bond formation, proton transfer, and subsequent functionalization can be addressed at the molecular level. Zintl chemistry therefore marks the point at which extended silicon frameworks begin to approach the chemically addressable intermediates characteristic of molecular silicon hydride chemistry [38, 51, 52, 53, 54].

**FIGURE 6:**
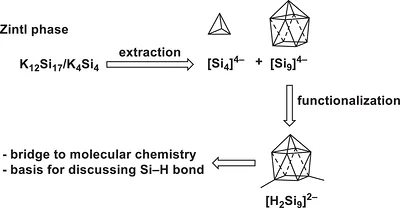
Zintl phases as a bridge from solid‐state to molecular silicon hydride chemistry. Preorganized polyanionic silicon units embedded in solid‐state Zintl phases can be transferred into solution and undergo further functionalization, including protonation, thereby providing a basis for molecularly addressable silicon species in which Si─H bond formation, proton transfer, and the elementary steps relevant to later hydrogen‐release chemistry can be discussed.

This transition is precisely what makes silicon Zintl phases so important for the development of molecular silicon hydride chemistry [38, 51, 53]. They demonstrate that extended silicon frameworks can generate discrete, solution‐accessible silicon clusters whose reactivity already resembles that of molecular systems [38, 51, 52, 53]. At the same time, this transformation from the solid‐state to the molecular regime is not universal, since not all Zintl phases dissolve under conditions that preserve preorganized silicon polyanions as discrete reactive species [37, 38, 51]. Beyond simple extraction chemistry, such Zintl‐derived silicon units can therefore also be regarded as chemically addressable precursors to siliconoids, linked cluster architectures, and related forms of silicon‐based materials chemistry [51, 52, 53, 54, 55]. The following chapter consequently turns to molecular systems, in which the elementary steps of Si─H activation and hydrogen release can be examined in a more isolated and mechanistically defined setting.

## 3 Molecular Compounds: Metal‐Mediated Systems

In contrast to the extended silicon environments discussed above, molecular metal‐mediated systems place Si─H bonds within well‐defined coordination spheres, thereby allowing the elementary steps of Si─H activation and hydrogen release to be analyzed in a far more controlled and mechanistically accessible manner. The focus of this chapter is therefore not transition‐metal organosilicon chemistry in its entirety, but specifically those activation modes that are mechanistically relevant to hydrogen evolution from silicon‐based systems. Broadly, these pathways can be divided into the classical activation modes of oxidative addition and redox‐neutral σ‐bond metathesis, and the nonclassical regimes of σ‐silane coordination, electrophilic activation, and cooperative heterolysis. A second and equally important aspect concerns the distinct hydrogen‐releasing pathways that may emerge from these activation manifolds [19].

### 3.1 Classical Si─H Activation: Oxidative Addition and σ‐Bond Metathesis

Oxidative addition represents one of the classical modes of Si─H bond activation and became the dominant mechanistic paradigm in early Pt‐group‐metal‐catalyzed hydrosilylation chemistry, as formulated in the Chalk–Harrod mechanism (Scheme 1) [19, 56, 57, 58, 59, 60].

**SCHEME 1:**
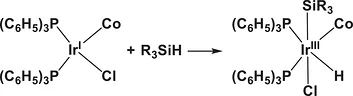
The classical oxidative addition pathway, as formulated by Chalk and Harrod for late transition metals, proceeds from Si─H coordination to the formation of hydrido–silyl complexes [56].

Mechanistically, the process begins with interaction of the Si─H bond with an electron‐rich low‐valent late transition metal, leading initially to formation of a σ‐complex, more accurately described in silicon chemistry as an η^2^‐silane complex [61, 62, 63, 64, 65, 66]. In such species, the Si─H bond remains formally intact while donating electron density into an empty metal orbital; concomitant back‐donation into the σ* orbital weakens the Si─H bond and facilitates cleavage [61, 62, 63, 64, 65, 66]. Subsequent oxidative addition generates a hydrido–silyl complex through formation of M─H and M─Si bonds, accompanied by an increase of both oxidation state and coordination number of the metal center [61, 62, 63, 64, 65, 66]. Further oxidative activation of additional Si─H bonds can extend this chemistry beyond classical hydrido–silyl complexes to molecular silylene and silicide species, illustrating the broad synthetic potential of oxidative Si─H activation [67]. In the context of hydrogen‐release chemistry, oxidative addition is therefore most important as a fundamental activation mode and mechanistic reference point rather than as the dominant direct pathway toward H_2_ evolution [61, 62, 63, 64, 65, 66, 67].

A second classical mode of Si─H activation is σ‐bond metathesis. This mechanism is most closely associated with high‐valent early d^0^ transition metals and related highly electropositive systems, although analogous bond‐exchange chemistry has also been discussed for selected additional frameworks [68, 69, 70, 71, 72, 73, 74, 75, 76, 77]. Within silicon chemistry, σ‐bond metathesis is particularly significant because it provides one of the clearest mechanistic connections between Si─H activation, H_2_ evolution, and Si─Si bond formation.

According to the mechanism proposed by Tilley (Scheme 2), dehydrogenative polymerization of hydrosilanes initiated by metal hydride complex **I** proceeds first through dehydrometalation via a four‐center σ‐bond‐metathesis‐type transition state (**II**), furnishing metal silyl complex **III** with concomitant release of H_2_.

**SCHEME 2:**
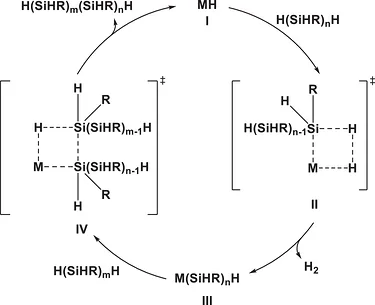
The redox‐neutral σ‐bond metathesis pathway characteristic of early d^0^ metals represents the mechanistic manifold most directly connecting Si─H activation with H_2_ evolution and Si─Si bond formation in silane dehydrocoupling, as proposed by Tilley [71].

In a subsequent step, **III** reacts with an additional hydrosilane through a second four‐center transition state (**IV**), thereby generating a new Si─Si bond and regenerating **I [** 71]. A representative example is provided by hydride complexes of the type CpCp^*^M(H)Cl (M = Zr, Hf) (**1**,**2**), which react with PhSiH_3_ (**3a**) through such a four‐center pathway to yield Cp_2_M(SiH_2_Ph)Cl (**4**,**5**) together with H_2_ evolution (Scheme 3) [70, 71].

**SCHEME 3:**
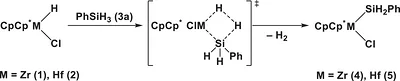
Reactions of d^0^ hydride complexes with hydrosilanes, as reported by Tilley [71].

These reactions are not merely stoichiometric curiosities but define the elementary bond‐exchange logic underlying early‐metal dehydrocoupling catalysis [68, 69, 70, 71, 72, 73, 74, 75, 76, 77]. Likewise, zirconocene‐derived systems such as Cp_2_Zr[Si(SiMe_3_)_3_]SiMe_3_ function as catalyst precursors for the dehydropolymerization of PhSiH_3_ to polysilanes with concomitant H_2_ elimination [70, 71]. In this sense, σ‐bond metathesis represents one of the most direct classical pathways connecting Si─H activation with hydrogen‐releasing Si─Si bond formation [68, 69, 70, 71, 72, 73, 74, 75, 76, 77, 78].

### 3.2 Nonclassical Si─H Activation: σ‐Silane Coordination, Electrophilic Activation, and Cooperative Heterolysis

The simple distinction between η^2^‐silane coordination and full oxidative addition is, however, frequently insufficient [79, 80, 81]. Many experimentally observed hydrosilane complexes occupy intermediate regions along a continuum between these limiting descriptions and are therefore more appropriately regarded as nonclassical Si─H activation states [79, 80, 81]. In such systems, the Si─H bond is neither merely coordinated nor fully cleaved, but substantially polarized, and this polarization can redirect reactivity away from the classical hydrido–silyl manifold [79, 80, 81]. The degree of partial activation directly influences both the electrophilicity at silicon and the extent of emerging M─H and M─Si bond character [79, 80, 81].

This continuum is exemplified by complexes such as [Cp(PMe_3_)_2_Ru(η^2^‐HSiCl_3_)]^+^ (**7**), the protonated form of Cp(PMe_3_)_2_RuSiCl_3_ (**6**), which is best understood as an intermediate case between simple silane coordination and complete oxidative addition (Scheme 4) [79, 80]. Such species demonstrate that Si─H activation is often more nuanced than a simple coordinated‐versus‐cleaved description would suggest [79, 80, 81].

**SCHEME 4:**
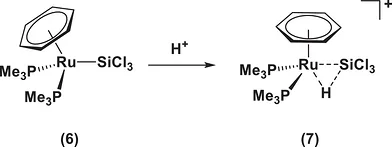
Electrophilic η^2^‐silane activation without full oxidative addition, as described by Lemke [79].

At the fully oxidative‐addition end of the spectrum, classical metal hydrido–silyl complexes predominate and generally behave as hydride donors, typically reverting through reductive silane elimination rather than direct H_2_ release [62, 63, 64, 65].

A distinct nonclassical pathway is the electrophilic activation of the Si─H bond [82, 83, 84, 85, 86, 87]. In η^2^‐silane complexes where coordination remains the dominant bonding motif, the silicon center can become sufficiently electrophilic to undergo nucleophilic attack without prior formation of a classical hydrido–silyl intermediate [82, 83, 84, 85, 86, 87]. This concept is particularly important in the silane alcoholysis chemistry reported by Crabtree, where an iridium system mediates H_2_ evolution through a pathway better described by electrophilic η^2^‐silane activation than by straightforward oxidative addition (Scheme 5) [82].

**SCHEME 5:**
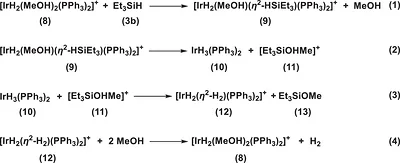
Electrophilic activation of Et_3_SiH via η^2^‐silane coordination, followed by nucleophilic attack, proton transfer, and H_2_ release in Crabtree's iridium‐catalyzed silane alcoholysis.

In the mechanism proposed by Crabtree, Et_3_SiH (**3b**) first displaces one MeOH ligand from complex **8** to generate η^2^‐silane complex **9** (Scheme 5, equation 1). Coordination to the electrophilic Ir^3+^ center strongly activates the Si─H bond toward nucleophilic attack by MeOH, leading ultimately to protonated silyl ether **11** and trihydride complex **10** (Scheme 5, equation 2). Protonation of **10** by **11** then affords dihydrogen hydride species **12** together with silyl ether **13** (Scheme 5, equation 3), while subsequent displacement of the coordinated H_2_ ligand in **12** by MeOH regenerates **8** and releases molecular hydrogen (Scheme 5, equation 4) [82]. In such systems, increased Lewis acidity at silicon becomes mechanistically decisive [82, 83, 84, 85, 86, 87]. Electrophilic activation is therefore especially relevant to alcoholysis, hydrolysis, and more generally Si─H/X–H dehydrogenative coupling reactions [15, 16, 82, 83, 84, 85, 86, 87].

A further nonclassical category comprises cooperative heterolysis, often described as metal–ligand cooperation or cooperative Si─H activation. In these systems, Si─H cleavage occurs heterolytically across a polarized metal–ligand bond or a closely cooperating metal/ligand pair. The mechanistic emphasis therefore shifts away from formal oxidative addition toward generation of polarized silicon‐ and hydrogen‐containing fragments that subsequently enter silyl‐transfer, Si─X bond‐forming, or dehydrogenative pathways [88, 89, 90, 91]. The decisive feature is not a specific metal class but the presence of a sufficiently polarized cooperative metal–ligand motif. Such behavior has consequently been observed across several platforms, including Ru, W, and Fe systems [88, 89, 90, 91]. Particularly important examples are the Ru–S and W/CO systems developed by Oestreich and coworkers as well as the Fe–amide chemistry reported by Thomas and coworkers [89, 90, 91].

The Ru–S system developed by Oestreich and coworkers demonstrated C3‐selective C─H silylation of indole **15** with hydrosilane **3c** to form **16**, [88] dehydrogenative coupling of ketone **17** with hydrosilane **3d** and of ketimine **19** with hydrosilane **3c** to afford **18** and **20**, respectively, [92, 93] as well as N‐silylation of indole **21** with hydrosilane **3e** to form **22** (Figure 7) [94].

**FIGURE 7:**
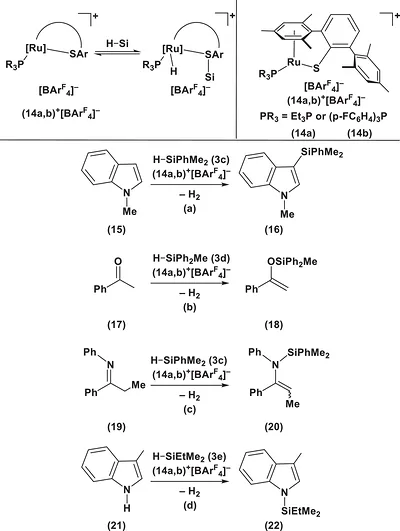
Top: Proposed cooperative Si─H bond heterolysis by ruthenium(II) thiolate complexes, as reported by Oestreich [89]. Bottom: Reported dehydrogenative catalytic applications: (a) C3‐silylation of indoles, [88] (b) dehydrogenative coupling of enolizable ketones, [92] (c) dehydrogenative coupling of enolizable ketimines, [93] and (d) N‐silylation of indoles [94].

The PNNP‐supported Fe(II) complex **23** reported by Thomas activates primary silanes across the Fe–amide bond in a metal–ligand cooperative fashion. Reaction with excess silane proceeds further to give dehydrogenatively coupled disilane‐type product **24** with concomitant H_2_ evolution (Scheme 6) [91]. Among these systems, the Ru–S and Fe–amide platforms are particularly relevant because they combine detailed mechanistic insight with experimentally demonstrated hydrogen‐release reactivity [88, 89, 91]. By contrast, the W/CO systems are especially instructive from a conceptual perspective, since they reveal that reactivity initially appearing oxidative‐addition‐like may in fact be more accurately described as cooperative heterolytic Si─H cleavage [90].

**SCHEME 6:**
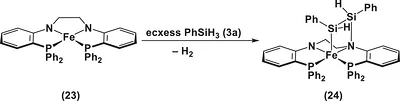
Reaction of bis(amido)bis(phosphine) iron(II) complex (PNNP)Fe (**23**) with an excess of PhSiH_3_ (**3a**) leads to dehydrogenative Si─Si coupling, as reported by Thomas [91].

### 3.3 Hydrogen Release Pathways in Metal‐Mediated Si─H Chemistry

The various modes of metal‐mediated Si─H activation represent mechanistic entry points that can lead to fundamentally different hydrogen‐releasing outcomes [19, 77, 85, 88]. Oxidative addition, σ‐bond metathesis, η^2^‐silane coordination, electrophilic activation, and cooperative heterolysis may all ultimately feed into dehydrogenative transformations, including Si─Si bond formation, Si─X (X = C, N, O, S) bond formation, and, in more specialized cases, H_2_ release from discrete complexes, either irreversibly or reversibly [19, 77, 85, 88].

Among these possibilities, Si─Si dehydrocoupling and dehydropolymerization constitute the most established H_2_‐releasing manifold in metal‐mediated silane chemistry [68, 69, 70, 71, 72, 73, 74, 75, 76, 77, 95, 96]. In these transformations, hydrogen is released overall as silanes undergo dehydrogenative coupling to form disilanes, oligomers, or polysilanes [68, 69, 70, 71, 72, 73, 74, 75, 76, 77, 95, 96]. Mechanistically, this chemistry is historically and conceptually most closely associated with early‐transition‐metal and lanthanide systems operating through redox‐neutral four‐center σ‐bond‐metathesis pathways [68, 69, 70, 71, 72, 73, 74, 75, 76, 77]. Foundational contributions from Tilley and coworkers on early d^0^‐metal dehydropolymerization, together with organolanthanide dehydrocoupling studies by Marks and coworkers, established that net H_2_‐releasing Si─Si bond formation can proceed entirely without classical oxidative addition [70, 71, 72].

Tilley and coworkers demonstrated, for example, that zirconocene complex **25** efficiently catalyzes dehydropolymerization of primary hydrosilanes such as PhSiH_3_ (**3a**) (Scheme 7) [71]. Subsequent studies further established that dehydrogenative Si─Si bond formation is not restricted to d^0^ or lanthanide systems, but can also arise in first‐row (Scheme 6) and late‐transition‐metal frameworks, for instance through cooperative Si─H activation or related nonclassical pathways [74, 91, 95, 96].

**SCHEME 7:**
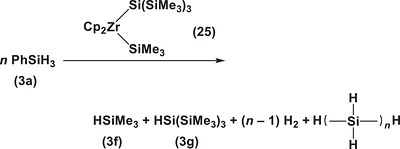
Dehydropolymerization of hydrosilanes using a zirconium(0) catalyst, as reported by Tilley [71].

Hydrogen release from silanes is, however, not limited to Si─Si bond formation. A second major reaction class is Si─H/X–H dehydrogenative coupling, in which reaction with a protic X–H substrate generates a new Si─X bond together with concomitant H_2_ evolution [15, 16, 82, 88, 89, 97, 98, 99, 100]. Within the present context, oxygen‐ and nitrogen‐based coupling partners are especially important, whereas carbon‐based examples are less central [15, 16, 97, 98, 99, 100]. Mechanistically, these reactions are often linked more closely to electrophilic σ‐silane activation or cooperative heterolytic cleavage than to classical oxidative‐addition pathways [82, 83, 84, 85, 86, 87, 88, 89, 97]. In such systems, H_2_ evolution arises less from a discrete metal‐centered elimination step than from polarized Si─H activation followed by Si─X bond formation and hydride/proton recombination [82, 88, 89, 97]. A historically important example again originates from Crabtree's silane alcoholysis chemistry, which demonstrated that efficient H_2_ evolution can emerge from electrophilic and nonclassical Si─H activation [82].

In the proposed mechanism, electrophilic activation of the Si─H bond generates iridium dihydrogen hydride complex **12** (Scheme 8, see also Scheme 5 and equations 1–3), from which displacement of the labile H_2_ ligand by methanol regenerates catalyst **8** while liberating molecular hydrogen (Scheme 8, see also Scheme 5 and equation 4) [82]. Complementary examples are provided by the Ru–S systems developed by Oestreich and coworkers, which likewise demonstrate net hydrogen liberation through cooperative heterolytic activation pathways (see Figure 7) [88, 89]. More recent studies have moved this chemistry toward application‐oriented hydrogen‐storage concepts. García, Mata, and coworkers described dehydrogenative coupling of hydrosilanes with alcohols as a potential LOHC‐type strategy for on‐demand hydrogen release. In this concept, a silane/alcohol substrate pair is converted into the corresponding silyl ether with concomitant liberation of molecular hydrogen using ruthenium catalyst **26** (Figure 8). Hydrogen is thus effectively stored in the Si─H and O─H bonds of a liquid substrate combination and released under mild conditions through a chemically well‐defined Si─O bond‐forming process [15, 16]. Related cobalt‐catalyzed variants reported by Hreczychno, Kuciński and coworkers as well as by Oña‐Burgos and coworkers further illustrate how H_2_ evolution can be coupled directly to the formation of synthetically valuable silicon‐containing products [98, 99, 100].

**SCHEME 8:**
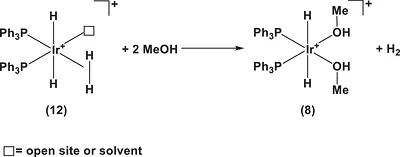
Displacement of a labile dihydrogen ligand by methanol, as reported by Crabtree [82].

**FIGURE 8:**
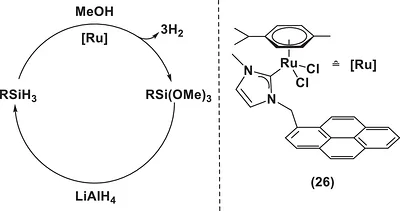
Dehydrogenative coupling of hydrosilanes with alcohols as a potential system for hydrogen storage and release, as presented by García and Mata [15].

A third, mechanistically distinct class comprises discrete metal complexes capable of releasing H_2_ at the level of a defined molecular species [101, 102, 103, 104]. In these systems, the focus shifts away from net dehydrogenative coupling chemistry toward equilibria between silane‐derived complexes and more unsaturated silicon‐containing intermediates [101, 102, 103, 104]. This behavior is illustrated particularly clearly by the reversible H_2_ elimination/addition processes associated with η^3^‐H_2_SiRR´/silylene interconversion in ruthenium chemistry (Scheme 9) [101].

**SCHEME 9:**
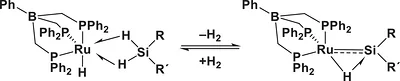
Reversible H_2_ elimination/addition involving η^3^‐H_2_SiRR´/silylene complexes, as described by Tilley [101].

Although this reactivity class represents a comparatively small subset of the broader dehydrocoupling literature, it is especially significant in the context of hydrogen‐release chemistry of silicon‐based systems because it conceptualizes H_2_ evolution as a chemically discrete and potentially reversible molecular process [101, 102, 103, 104].

## 4 Molecular Compounds: Metal‐Free Systems

Moving from metal‐mediated Si─H activation to fully metal‐free molecular systems, hydrogen release from silicon compounds can broadly be organized into three mechanistically distinct, though partially interconnected, manifolds: borane‐mediated electrophilic activation, a smaller class of non‐borane cationic electrophiles, and donor‐ or base‐assisted pathways involving higher‐coordinate silicon intermediates or transition states [17, 18, 19, 86, 87, 105, 106]. In contrast to the predominantly redox‐based activation patterns encountered in transition‐metal chemistry, these systems are governed primarily by polarization of the Si─H bond, nucleophilic attack at electrophilically activated silicon centers, and proton–hydride coupling processes that may, but do not necessarily, culminate in H_2_ release [18, 19, 86, 87, 105, 106].

### 4.1 Borane‐Mediated Si─H Activation and Dehydrogenative Coupling

Within this metal‐free landscape, the most established electrophilic route to hydrogen release is the borane‐mediated dehydrogenative coupling of hydrosilanes with protic X–H partners, particularly in the chemistry of B(C_6_F_5_)_3_ [105, 106]. Foundational work by Piers and coworkers demonstrated that B(C_6_F_5_)_3_ catalyzes the coupling of hydrosilanes with primary, secondary, tertiary, and phenolic alcohols to form silyl ethers with H_2_ as the sole stoichiometric byproduct [107].

Mechanistically, these reactions are best understood in terms of electrophilic activation of the Si─H bond in **I** by B(C_6_F_5_)_3_, generating a strongly polarized silane–borane adduct **II**. Upon addition of alcohol **III**, this activation evolves into a silylium‐like situation in which the silicon center becomes susceptible to nucleophilic attack by the alcohol **IV**, ultimately leading to Si─O bond formation, proton–hydride coupling, and catalyst regeneration to furnish silyl ether **V** (Scheme 10) [106, 107, 108].

**SCHEME 10:**
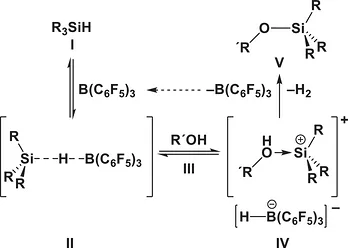
B(C_6_F_5_)_3_‐catalyzed dehydrogenative Si─O coupling of alcohols with silanes, as reported by Piers [107].

Importantly, this borane‐mediated Si─O coupling manifold extends well beyond small‐molecule alcoholysis chemistry. Brook, Grande, and Ganachaud, as well as Cella and Rubinsztajn, demonstrated that the same electrophilic activation principle can be transferred to polymer and materials chemistry, including the formation of polyaryloxysilanes and siloxanes from hydrosilanes and bisphenols under B(C_6_F_5_)_3_ catalysis [109, 110]. These studies are significant not because they fundamentally alter the mechanistic picture, but because they establish borane‐mediated Si─O coupling with concomitant H_2_ evolution as a broadly general and reproducible class of reactivity within metal‐free silicon chemistry [107, 109, 110].

Electrophilic borane activation is, however, not confined to Si─O bond formation. Rosenberg and coworkers extended this concept to Si─S dehydrocoupling chemistry by showing that B(C_6_F_5_)_3_ catalyzes the coupling of tertiary silanes (**3b**), disilanes (**3h**), and even poly(phenylsilane) with alkyl‐ and arylthiols (**27**,**29**) under transition‐metal‐free conditions, accompanied by H_2_ evolution and notably high chemoselectivity for Si─H over Si─Si activation (Scheme 11) [111].

**SCHEME 11:**
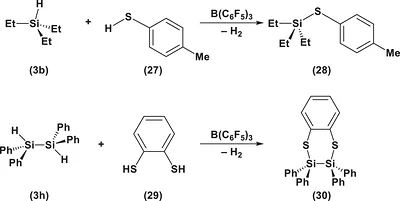
Heterodehydrogenative Si─S coupling of monosilanes (top) and disilanes (bottom) with thiols, as presented by Rosenberg [111].

A related extension toward Si─N bond formation was reported by Paradies and coworkers, who demonstrated that B(C_6_F_5_)_3_ promotes dehydrogenative coupling of hydrosilanes with anilines and carbazoles [112]. In the case of aniline **31**, reaction with silane **3d** proceeds through intermediate **32**, ultimately affording Si─N‐coupled product **33** with H_2_ as the sole byproduct (Scheme 12) [112].

**SCHEME 12:**
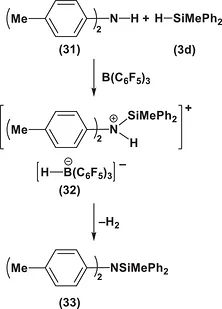
B(C_6_F_5_)_3_‐mediated dehydrogenative Si─N coupling, as reported by Paradies [112].

For indoles, however, the same activation manifold leads to more complex reaction outcomes. In these systems, the initially accessible dehydrocoupling pathway does not terminate at Si─N bond formation, but instead evolves into a combined Si─N bond‐forming and hydrogenation sequence that ultimately furnishes 1‐silylated indolines (Scheme 13) [112].

**SCHEME 13:**
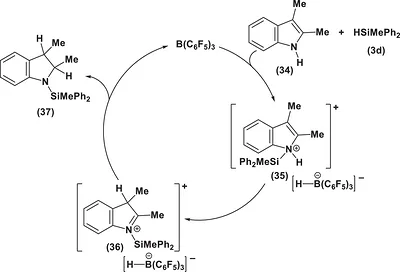
Mechanistic cycle for the Si─N formation and hydrogenation sequence according to Paradies [112].

Here, B(C_6_F_5_)_3_ first catalyzes Si─N coupling between indole **34** and hydrosilane **3d**. Rather than undergoing direct H_2_ elimination from intermediate **35**, the system proceeds through a [1, 5]‐sigmatropic rearrangement to generate **36**, after which the hydroborate fragment reduces the indole moiety to furnish indoline **37** while regenerating the catalyst [112].

This divergence in reactivity is mechanistically important because it highlights that electrophilic Si─H activation and H_2_ release are closely related, yet fundamentally distinct, outcomes. Ingleson and coworkers illustrated this point particularly clearly in B(C_6_F_5_)_3_‐mediated heteroarene functionalization, where dehydrosilylation, hydrosilylation, and hydrogenation compete while emerging from a common borane–silane activation manifold [113]. Likewise, Gevorgyan, Yamamoto, and coworkers showed in related borane‐catalyzed reduction and ether‐cleavage chemistry that electrophilic silane activation can readily divert reactivity toward C─O bond reduction rather than formal dehydrocoupling [114]. Even within the best‐developed borane systems, the decisive mechanistic issue is therefore not merely whether Si─H activation occurs, but whether the activated intermediate proceeds through productive proton–hydride coupling to liberate H_2_ instead of entering competing reduction or hydrosilylation pathways [106, 112, 113, 114, 115].

### 4.2 Electrophilic Activation and Chemoselectivity in Si─H Transformations

Beyond borane Lewis acids, a smaller but mechanistically highly instructive class of cationic main‐group electrophiles demonstrates that dehydrogenative Si─X coupling is not unique to boron‐based systems [86, 87]. Particularly informative are the electrophilic phosphonium cations developed by Stephan and coworkers. Their fluorophosphonium system promotes dehydrocoupling of hydrosilanes with amines, phenols, thiols, and carboxylic acids under metal‐free conditions, accompanied by H_2_ evolution and Si─E bond formation [116]. As in the borane systems, steric effects are decisive: smaller silanes react readily, whereas more hindered silanes react sluggishly or remain unreactive, consistent with a mechanism involving formation of a higher‐coordinate silicon intermediate following initial electrophilic activation of the Si─H bond [116].

At the same time, these phosphonium systems provide particularly clear insight into the chemoselectivity landscape of electrophilic Si─H activation. Stephan and coworkers further demonstrated that, in the presence of suitable olefins, the same activation manifold can be redirected from H_2_ release toward transfer hydrogenation chemistry [116].

As proposed by Stephan and coworkers, electrophilic fluorophosphonium cation, FP(C_6_F_5_)_3_^+^ (**38**), first activates the Si─H bond of the hydrosilane **II**, generating intermediate **III** and enabling nucleophilic attack by the amine **IV** (Figure 9). Subsequent transformations lead through intermediates **V** and **VI** to the dehydrocoupled Si─N product **VII** with concomitant H_2_ release. In the presence of an olefin **VIII**, however, this pathway becomes diverted: instead of liberating free H_2_, transient protonated intermediate **VI** transfers a proton to the olefin **VIII**, generating carbocation **X**, which is subsequently reduced by a phosphonium‐derived hydride to furnish hydrogenated olefin **XI** while regenerating catalyst **38** (Figure 9) [116].

**FIGURE 9:**
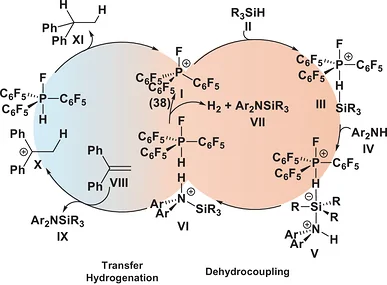
Proposed mechanism for the dehydrocoupling of amines and silanes, including the competing transfer hydrogenation of olefins, as proposed by Stephan [116].

Viewed from this perspective, dehydrocoupling and transfer hydrogenation do not constitute fundamentally separate mechanistic domains, but rather competing outcomes arising from a shared electrophilic Si─H activation manifold [116].

A related but mechanistically distinct picture emerged from our previously reported phosphonium systems (Scheme 14). We showed that protonated hydrosilyl phosphonium ions such as **39** undergo dehydrogenative cyclization to form **40**, whereas subtle changes in the reaction medium redirect the outcome from H_2_ evolution toward protodesilylation, yielding **41** (Scheme 14) [117]. This system is particularly instructive because it demonstrates, in a chemically discrete setting, how narrowly productive hydrogen release is separated from nonproductive pathways that consume the activated silicon species [117].

**SCHEME 14:**
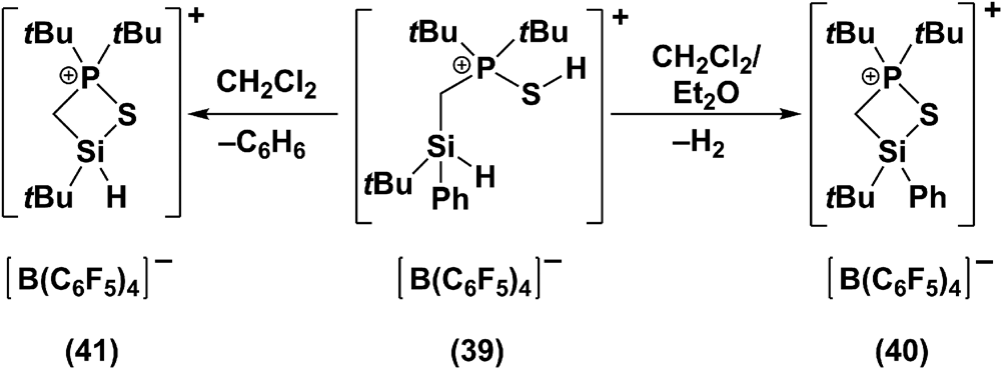
Solvent‐induced chemoselectivity switching in dehydrogenative Si─S cyclization, as reported by Bauer [117].

Subsequent structural and electronic studies further clarified how donor type and substituent pattern influence stabilization of these intramolecularly stabilized heterocyclic silyl phosphonium cations [117, 118]. Recent work has expanded the scope of non‐borane main‐group Si─H activation beyond phosphonium chemistry. Hejda, Hupf, Beckmann and coworkers showed that Lewis‐superacidic tellurenyl cations activate a wide range of hydrosilanes, including SiH_4_, enabling catalytic quinone reduction and silylation while providing detailed mechanistic insight into the underlying tellurium redox cycle [119, 120]. Similarly, Inoue and coworkers reported hydrosilane activation by a neutral germanium‐centered Lewis superacid, extending main‐group Si─H activation to germanium‐based Lewis superacids and catalytic hydrosilylations [121]. More broadly, the chemistry of strongly electrophilic silicon‐based Lewis acids has expanded substantially in recent years, although much of this work is more directly relevant to bond activation and catalysis in general than to hydrogen release specifically. Greb and coworkers established the pronounced Lewis acidity of bis(catecholato)silanes and demonstrated how systematic modification of the catecholate framework can drive neutral silicon Lewis acidity into the superacidic regime [122]. They further showed that bis(perchlorocatecholato)silane forms ring‐strain‐enabled hidden frustrated Lewis pairs with suitable bidonors, [123] while Inoue and coworkers introduced bis(perfluoropinacolato)silane as another neutral silicon Lewis superacid capable of Si─F bond activation [124]. Hermannsdorfer and Driess subsequently demonstrated that Si(OTf)_4_ functions as a neutral hard and soft silicon Lewis superacid, [125] and Greb and coworkers finally showed that a neutral intermolecular frustrated Lewis pair composed of a silicon(IV) species and an amine can activate H_2_ under carefully tuned conditions [126]. Collectively, these studies establish strongly electrophilic silicon‐centered Lewis acidity as a genuine and increasingly versatile design platform rather than a merely unusual chemical curiosity [122, 123, 124, 125, 126].

### 4.3 Donor‐Assisted Si─H Activation via Higher‐Coordinate Silicon Intermediates

A second major metal‐free route to hydrogen release proceeds not through external electrophilic activation, but through donor‐ or base‐assisted Si─H activation directly at silicon, frequently involving higher‐coordinate silicon intermediates or transition states [105, 127, 128]. Early studies by Corriu and coworkers on Si─N bond formation already indicated that nucleophilic attack at tetravalent hydrosilanes can generate reactive higher‐coordinate silicon species from which Si─N and Si─O bond formation, together with H_2_ release, become accessible (Scheme 15) [129].

**SCHEME 15:**
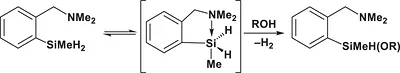
Donor‐assisted formation of a higher‐coordinate silicon species in the context of H_2_‐releasing Si─O bond formation, adapted from the nucleophilic Si─H activation chemistry reported by Corriu [129].

More generally, the broader literature on penta‐ and hexacoordinate silicon compounds, comprehensively reviewed by Corriu and coworkers, established a conceptual framework in which such species came to be viewed not merely as structural curiosities, but as plausible intermediates in substitution and bond‐activation chemistry at silicon [127, 128, 130].

The alcoholysis studies reported by Corriu and coworkers illustrate particularly clearly that pentacoordinate hydridosilicates are not static structural motifs, but chemically active species capable of further substitution chemistry at silicon. Alcoholysis of [HSi(OMe)_4_]^–^ (**42**) with MeOH formally generates pentacoordinate silicate [Si(OMe)_5_]^–^ (**44**) with concomitant H_2_ evolution, although **44** rapidly decomposes to Si(OMe)_4_ (**43**) unless stabilized by 18‐crown‐6 ether (Scheme 16, equations 5 and 6) [130]. Likewise, alcoholysis of dihydridosilicate [H_2_Si(OPr*^i^*)_3_]^–^ (**45**) with *iso*‐propanol proceeds through nucleophilic substitution at pentacoordinate silicon via a hexacoordinate transition state to yield [HSi(OPr*^i^*)_4_]^–^ (**46**) (Scheme 16, equation 7) [130].

**SCHEME 16:**
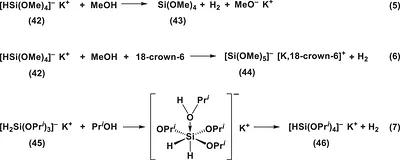
Alcoholysis of pentacoordinate hydridosilicates, as presented by Corriu [130].

In many classical cases, pentacoordinate silicon compounds adopt trigonal‐bipyramidal geometries and undergo configurational interchange through Berry pseudorotation or related permutational processes, as later demonstrated particularly clearly by Lammertsma and coworkers in stable pentaorganosilicates [131, 132]. This donor‐assisted activation logic became especially important in dehydrogenative Si─O coupling chemistry. Oestreich and coworkers showed that potassium *tert*‐butoxide catalyzes dehydrogenative coupling of alcohols with hydrosilanes through intervention of a higher‐coordinate silicon intermediate in which nucleophilic attack by alkoxide activates the Si─H bond toward H_2_ elimination (Scheme 17) [133].

**SCHEME 17:**
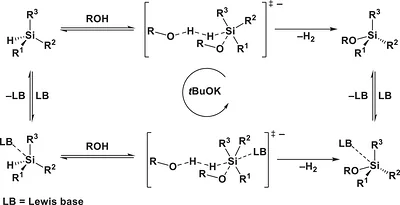
Proposed mechanism for donor‐assisted dehydrogenative Si─O coupling according to Weickgenannt and Oestreich [133].

They later extended this base‐promoted Si─O coupling chemistry to dihydrosilanes and diols, in which both hydroxy groups of the diol are linked by a R_2_Si unit to form cyclic silylene‐protected diols with H_2_ as the only stoichiometric byproduct [134]. Subsequent studies broadened the operational scope of this activation manifold further. Grubbs and coworkers demonstrated sodium hydroxide catalysis under remarkably mild conditions, [135] while Vannucci and coworkers and Pawluć and coworkers showed that K_2_CO_3_ and NaHB(*sec*‐Bu)_3_ likewise promote dehydrogenative Si─O coupling in suitable systems [136, 137]. Collectively, these studies establish that H_2_‐releasing Si─O coupling can emerge from multiple classes of donor‐assisted activation pathways [133, 134, 135, 136, 137].

More recently, Han, Chen, and coworkers reported the phenylphosphonic acid‐catalyzed dehydrogenative coupling of hydrosilanes with alcohols under solvent‐free conditions, further expanding the scope of metal‐free H_2_‐releasing Si─O coupling [138]. Subsequently, Martín and Sayari demonstrated room‐temperature H_2_ generation from hydrosilanes and amino alcohols without the need for an added catalyst, with the amine functionality serving as an internal promoter of the dehydrogenative coupling [139]. Earlier investigations by Schowen and coworkers on hydride expulsion from silicon had already suggested a transition state in which proton donation to the departing hydride develops while Si─H cleavage remains incomplete, consistent with a proton–hydride coupling picture rather than simple hydride dissociation [140]. A more explicit modern interpretation was later provided by Belkova, Shubina and coworkers through combined spectroscopic and DFT studies on base‐catalyzed dehydrocoupling of phenylsilanes with alcohols [141].

Their work demonstrated that coordination of a Lewis base (RO^–^) to silicon substantially increases silane hydricity and that tertiary complexes of the type [R_4−n_Si(Lewis base)H_n_]···HOR´ are crucial intermediates for efficient proton–hydride transfer and H_2_ evolution, with alkoxides emerging as particularly effective activators (Scheme 18) [141]. A conceptually related intramolecular variant was subsequently reported by Durka, Luliński, and coworkers, who showed that ortho‐donor substitution in arylsilanes can promote Si─H activation and, under suitable conditions, H_2_ evolution through internally assisted pathways [142]. Together, these findings strongly support the view that donor coordination can transform otherwise only moderately reactive silanes into substantially more hydridic species capable of productive proton–hydride coupling [140, 141, 142].

**SCHEME 18:**
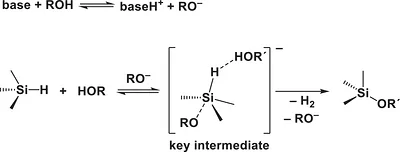
Formation of the key intermediate as a critical prerequisite for Si─O dehydrocoupling of hydrosilanes and alkoxides, as proposed by Belkova and Shubina [141].

Related donor‐assisted Si─H activation has also found application beyond H_2_‐release chemistry. Greb, Inoue, and coworkers recently demonstrated that silyliumylidene‐derived silicon species promote catalytic CO_2_ hydrosilylation through transient higher‐coordinate silicon intermediates, channeling Si─H activation into hydride transfer rather than H_2_ evolution [143]. At the same time, the literature on isolable hydridosilicates makes equally clear that higher coordination alone is not sufficient to guarantee hydrogen evolution. Steed and coworkers reported the first structurally characterized higher‐coordinate silicon hydride, and subsequent studies expanded this chemistry to additional hydridosilicates displaying trigonal‐bipyramidal geometries, axial hydrides, hydride bridging, and pronounced reactivity under rigorously anhydrous conditions [144, 145]. Earlier work by Corriu and coworkers had already established pentacoordinate hydridosilicates as chemically versatile species capable of nucleophilic substitution, hydride transfer, alcoholysis, and even one‐electron processes, thereby emphasizing their mechanistic relevance in silicon chemistry [146]. Okuda and coworkers further connected higher‐coordinate hydrosilicates with alkali‐metal amide frameworks and catalytic reactivity, [147] while also isolating a macrocycle‐supported hexahydridosilicate that expanded the known structural range of discrete higher‐coordinate silicon hydrides [27]. However, such compounds are generally highly reactive and do not, by themselves, constitute controlled H_2_‐release systems [27, 144, 145, 146, 147].

A particularly instructive example was provided by Greb and coworkers. Their calix[4]pyrrole hydridosilicate possesses an almost square‐pyramidal coordination environment with a short apical Si─H bond, yet displays unusual water stability and markedly diminished hydridic reactivity compared with previously known hydridosilicates [148]. Thus, even an anionic higher‐coordinate silicon hydride does not necessarily function as a facile H_2_‐releasing intermediate [148]. This conclusion is reinforced by subsequent studies from Greb and coworkers describing additional higher‐coordinate silicon anions stabilized by weak donors, which confirmed the mechanistic relevance of such species in Lewis‐base catalysis while simultaneously revealing that their structural and electronic consequences are considerably subtler than earlier empirical models had suggested [149].

Accordingly, donor‐ and base‐assisted hydrogen release in metal‐free silicon chemistry is best understood in terms of higher‐coordinate‐silicon‐enabled reactivity rather than higher coordination as a sufficient cause in itself [127, 128, 129, 130, 131, 132, 133, 134, 135, 136, 137, 140, 141, 142, 144, 145, 146, 147, 148, 149]. The available literature collectively indicates that productive H_2_ evolution depends on the interplay of donor identity, ligand dynamics, ion pairing, and, crucially, the ability of the activated silicon center to access the specific proton–hydride arrangement required for H_2_ elimination [28, 131, 132, 141, 142, 144, 145, 146, 147, 148, 149].

## 5 Geometric and Dynamic Control of H_2_ Release From Higher‐Coordinate Silicon

As discussed above, donor‐ and base‐assisted formation of higher‐coordinate silicon species provides a viable pathway toward metal‐free H_2_ release in silicon‐based systems [133, 134, 135, 136, 137, 140, 141, 142]. At the same time, higher coordination alone cannot be regarded as a sufficient prerequisite for hydrogen evolution. Earlier mechanistic studies by Schowen and coworkers as well as by Eaborn and Jenkins already interpreted base‐assisted Si─H/H_2_ chemistry in terms of pentacoordinate silicon intermediates and concerted proton transfer to the departing hydride, thereby suggesting that productive H_2_ formation depends not simply on Si─H activation, but on access to a specific reactive arrangement, [150, 151] a concept that is consistent with recent computational studies emphasizing structural flexibility as a key mechanistic parameter also in silicon‐mediated H_2_ activation [152]. The decisive mechanistic question is therefore not simply whether a higher‐coordinate silicon species can form, but whether its structural organization and fluxional behavior enable the corresponding higher‐coordinate framework to access the reactive geometry required to transform Si─H activation into actual H─H bond formation and subsequent H_2_ release 28.

The relevant structural space is best viewed as a continuum between trigonal‐bipyramidal and square‐pyramidal coordination geometries, since both hydride position and overall distortion of the coordination sphere can become mechanistically decisive [127, 128, 129, 131, 132]. Structural investigations by Steed and coworkers demonstrated experimentally that axial hydride occupation in trigonal‐bipyramidal hydridosilicates (**47**) is a realistic and accessible structural motif (Figure 10a) [144, 145]. More broadly, the higher‐coordinate silicon chemistry established by Corriu and coworkers, together with the configurational analyses of pentacoordinate silicates (**48**, **49**) by Lammertsma and coworkers (Figure 10b), highlights that trigonal‐bipyramidal/square‐pyramidal distortions, site preferences, and Berry‐type ligand permutation processes are not merely structural descriptors, but mechanistically relevant features governing silicon reactivity [128, 129, 130, 131, 132, 144, 145].

**FIGURE 10:**
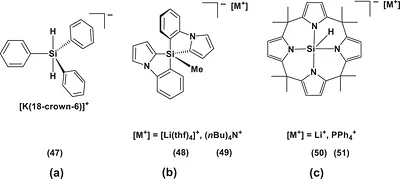
Stable pentacoordinate silicates: (a) higher‐coordinate silicon hydride in trigonal‐bipyramidal geometry with hydrides in axial positions, as reported by Steed, [144] (b) higher‐coordinate silicate in trigonal‐bipyramidal geometry, as reported by Lammertsma, [131] and (c) higher‐coordinate silicate in a nearly square‐pyramidal geometry with hydride in the apical position, as reported by Greb [148].

A particularly instructive distinction between productive and nonproductive higher‐coordinate silicon hydrides emerges from the work of Greb and coworkers. In their neutral intermolecular silicon(IV)‐amine frustrated Lewis pair system, H_2_ activation furnishes an isolable hydridosilicate species [126]. While this demonstrates that hydridosilicate formation following H_2_ cleavage is entirely feasible, no productive H_2_‐release pathway originates from the resulting intermediate [126]. These findings therefore indicate that H_2_ activation and H_2_ elimination cannot simply be treated as reverse manifestations of the same process, and that the mere presence of a higher‐coordinate silicon hydride is insufficient to account for hydrogen evolution [126]. A related but even more strongly stabilized example is the calix[4]pyrrole hydridosilicate (**50**, **51**) reported by Ebner and Greb (Figure 10c), which features an almost square‐pyramidal silicon environment, a short apical Si─H bond, and markedly diminished hydridic reactivity [148]. In this case, the higher‐coordinate silicon hydride behaves as a persistent endpoint rather than a productive intermediate [148].

Against this background, our recent work provides direct insight into the geometric and dynamic requirements for productive H_2_ release [28] (Scheme 19). In the Dipp‐substituted diaminohydridocyclosilane **52**, variable‐temperature NMR spectroscopy combined with computational analysis supports the involvement of pentacoordinate silicon intermediates in which both equatorial (**52a**) and axial (**52b**) hydride isomers are accessible within a trigonal‐bipyramidal framework (Scheme 19). Productive H_2_ evolution and formation of **53** were found to depend critically on dynamic permutational isomerism, which enables access to the axial hydride arrangement required for elimination. In this way, Berry‐type pseudorotation becomes a mechanistically essential process linking higher coordination to productive hydrogen release and establishing a foundation for metal‐free dehydrogenative coupling chemistry [28].

**SCHEME 19:**
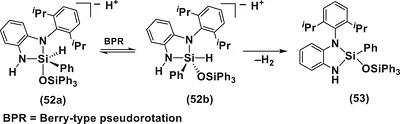
A higher‐coordinate hydridosilane capable of accessing a hydrogen‐releasing geometry through Berry‐type pseudorotation, as reported by Bauer [28].

These findings demonstrate that higher coordination alone does not guarantee H_2_ release. Rather, productive hydrogen evolution requires a dynamically accessible silicon framework capable of reaching the specific reactive hydride geometry necessary for H─H bond formation and elimination [28].

## 6 Summary and Outlook

Hydrogen release from silicon–hydrogen systems spans a broad structural and mechanistic continuum, ranging from extended solid‐state materials to discrete molecular compounds (Table 1). Across these regimes, Si─H activation and H_2_ evolution are not governed by a single universal pathway, but instead arise from the interplay of structure, electronic polarization, coordination environment, and the dynamic accessibility of reactive geometries.

**TABLE 1:** Comparison of hydrogen‐release mechanisms across silicon–hydrogen systems.

System	Si─H Environment	Activation	Chemical Outcome
Crystalline silicon surfaces	Ordered surface monohydrides, hydrogenated dimers, and localized higher‐hydride motifs	Thermally activated recombination of neighboring surface hydrides, coupled to surface rearrangement	H_2_ desorption and reconstruction of the silicon surface
Nanoporous and amorphous silicon	Heterogeneous SiH_x_ populations distributed across pores, defects and disordered networks	Recombination, hydrogen diffusion and network relaxation	Thermally or chemically induced hydrogen release
Silicon Zintl phases and clusters	Polyanionic Si_4_ and Si_9_ units and their protonated or functionalized derivatives	Cluster extraction, protonation, rearrangement, and functionalization	Formation of discrete molecular silicon species; no general direct H_2_ release pathway established
Metal‐mediated molecular systems	η^2^‐Silane complexes, hydrido–silyl species, metal hydrides, and cooperatively activated Si─H bonds	Oxidative addition, σ‐bond metathesis, electrophilic activation, and metal–ligand cooperation	Si─Si or Si─X bond formation with H_2_ evolution
Metal‐free electrophilic systems	Polarized silane–borane adducts, silylium‐like intermediates and activated hydrosilanes	Nucleophilic attack at electrophilically activated silicon	Si─O, Si─N, or Si─S bond formation with H_2_ evolution
Donor‐assisted higher‐coordinate silicon systems	Pentacoordinate or hexacoordinate silicon intermediates with differently positioned hydrides	Donor‐induced Si─H polarization, ligand permutation, and proton‐hydride coupling	H_2_ releasing dehydrocoupling or, alternatively, hydride transfer and substitution

In extended silicon materials such as crystalline surfaces, nanoporous silicon, and amorphous networks, hydrogen release is governed by structure‐dependent recombinative processes rather than by simple Si─H bond cleavage. Key factors include local surface geometry, hydrogen coverage, and the ability of the silicon framework to reorganize during desorption. Zintl phases further broaden this landscape by bridging solid‐state silicon chemistry and molecular reactivity, as polyanionic silicon units can be transferred into solution and subsequently undergo protonation and functionalization, thereby providing access to discrete Si─H chemistry.

In molecular systems, the mechanistic landscape becomes more defined yet remains highly diverse. Metal‐mediated pathways encompass oxidative addition, σ‐bond metathesis, electrophilic activation, and cooperative heterolysis, whereas metal‐free systems primarily rely on electrophilic or donor‐assisted Si─H activation. In both cases, hydrogen evolution most commonly occurs through dehydrocoupling reactions forming Si─Si or Si─X bonds, although competing pathways such as hydrosilylation, transfer hydrogenation, and bond rearrangement frequently coexist.

A recurring mechanistic theme across all classes of silicon‐based systems is the involvement of higher‐coordinate silicon species. However, higher coordination alone is insufficient to ensure productive H_2_ release. Instead, the accessibility of specific reactive geometries, particularly axial hydride arrangements within fluxional trigonal‐bipyramidal frameworks, together with dynamic permutational isomerism, emerges as a decisive prerequisite for H─H bond formation and hydrogen elimination. Geometric flexibility and coordination dynamics therefore appear to be central design principles in silicon‐based hydrogen chemistry.

Beyond hydrogen release, the same mechanistic principles may provide a broader basis for catalytic bond‐forming processes at silicon. Higher‐coordinate silicon species occur as reactive intermediates or catalyst‐associated states in dehydrogenative Si─O and Si─N bond formation, hydrosilylation, and Lewis‐base‐catalyzed substitution chemistry. Together, these examples suggest that donor identity, ion pairing, coordination geometry, and ligand dynamics govern not only Si─H activation itself, but also whether the resulting intermediate proceeds toward H_2_ evolution, hydride transfer, or Si─X bond formation. Geometry‐controlled access to higher‐coordinate silicon species may therefore represent a general strategy for directing catalytic reactivity beyond hydrogen release.

A central challenge is therefore not simply to promote Si─H activation, but to identify and control the productive higher‐coordinate intermediates that govern reaction outcome. Key questions concern their lifetime, the rate of geometric interconversion, and whether permutation or the subsequent H─H‐forming step determines reactivity and selectivity. Addressing these challenges will require closer integration of in situ spectroscopy, kinetic analysis, computation, and structurally defined model systems. An equally important objective will be to translate these molecular mechanistic principles into extended silicon materials, thereby establishing common design concepts across molecular and solid‐state hydrogen‐release systems. More broadly, achieving this goal may enable the development of robust catalytic systems and practical silicon‐based hydrogen‐storage technologies, representing an important long‐term objective for the field.

Overall, silicon‐based hydrogen‐release chemistry occupies a unique position at the interface of materials science, organometallic chemistry, and main‐group reactivity. Continued advances in this field are expected not only to refine strategies for hydrogen storage and release, but also to deepen our understanding of how bond activation is governed by structure, coordination, and molecular dynamics. Future progress will likely depend on the rational design of silicon coordination environments through electronic tuning, steric control, and cooperative donor interactions. Ultimately, connecting H_2_ activation in silicon‐based frustrated Lewis pair systems with geometry‐controlled H_2_ evolution in higher‐coordinate silicon hydrides may provide the basis for a unified mechanistic framework in which hydrogen uptake and release are governed by the same underlying dynamic principles.

## Conflicts of Interest

The authors declare no conflicts of interest.

## Data Availability

Data sharing not applicable to this article as no datasets were generated or analyzed during the current study.
